# The Role of Host Gender in the Pathogenesis of *Cryptococcus neoformans* Infections

**DOI:** 10.1371/journal.pone.0063632

**Published:** 2013-05-31

**Authors:** Erin E. McClelland, Letizia M. Hobbs, Johanna Rivera, Arturo Casadevall, Wayne K. Potts, Jennifer M. Smith, Jeramia J. Ory

**Affiliations:** 1 The Commonwealth Medical College, Department of Basic Sciences, Scranton, Pennsylvania, United States of America; 2 Albert Einstein College of Medicine, Department of Microbiology and Immunology, Bronx, New York, United States of America; 3 University of Utah, Department of Biology, Salt Lake City, Utah, United States of America; 4 King's College, Department of Biology, Wilkes-Barre, Pennsylvania, United States of America; 5 Middle Tennessee State University, Department of Biology, Murfreesboro, Tennessee, United States of America; 6 Musculoskeletal Transplant Foundation, Microbiology Laboratory, Jessup, Pennsylvania, United States of America; The University of Texas at San Antonio, United States of America

## Abstract

*Cryptococcus neoformans* (*Cn*) is a pathogenic yeast and the cause of cryptococcal meningitis. Prevalence of disease between males and females is skewed, with males having an increased incidence of disease. Based on the reported gender susceptibility differences to *Cn* in the literature, we used clinical isolates from Botswanan HIV-infected patients to test the hypothesis that different gender environments exerted different selective pressures on *Cn*. When we examined this data set, we found that men had significantly higher risk of death despite having significantly higher CD4^+^ T lymphocyte counts upon admittance to the hospital. These observations suggested that *Cn* strains are uniquely adapted to different host gender environments and that the male immune response may be less efficient in controlling *Cn* infection. To discriminate between these possibilities, we tested whether there were phenotypic differences between strains isolated from males and females and whether there was an interaction between *Cn* and the host immune response. Virulence phenotypes showed that *Cn* isolates from females had longer doubling times and released more capsular glucoronoxylomannan (GXM). The presence of testosterone but not 17-β estradiol was associated with higher levels of GXM release for a laboratory strain and 28 clinical isolates. We also measured phagocytic efficiency, survival of *Cn,* and amount of killing of human macrophages by *Cn* after incubation with four isolates. While macrophages from females phagocytosed more *Cn* than macrophages from males, male macrophages had a higher fungal burden and showed increased killing by *Cn*. These data are consistent with the hypothesis that differential interaction between *Cn* and macrophages within different gender environments contribute to the increased prevalence of cryptococcosis in males. This could be related to differential expression of cryptococcal virulence genes and capsule metabolism, changes in *Cn* phagocytosis and increased death of *Cn*-infected macrophages.

## Introduction

*Cryptococcus neoformans* (*Cn*) is a pathogenic yeast that is the causative agent of cryptococcosis, a life-threatening fungal disease that affects the central nervous system. The frequency of *Cn* infections has increased exponentially in the last 30 years, mainly due to the HIV epidemic, but also to the increasing use of immunosuppressive therapies for organ transplantation and chemotherapy [3]. *Cn* epidemiology reveals a large discrepancy in the incidence of *Cn* infections in male and female patients (including AIDS and organ transplant patients), with males having a higher incidence of *Cn* infection than females. Interestingly, the gender susceptibility difference in *Cn* infections was noted even before the start of the HIV epidemic, where the incidence of *Cn* infection was 2–3:1 males:females [4]–[6]. Both prior to the HIV epidemic and recently, a common explanation for the increased incidence in males was increased exposure to *Cn* [7]–[9]. However, antigen reactivity studies suggest that males and females are exposed at equal rates [9], [10]. Previous reports show that estrogen inhibits growth of *Cn in vitro* [9] and that the administration of diethylstilbestrol to *Cn* patients increases the activity of patient leukocytes to phagocytose *Cn.* [11], [12]. Fungaemia, disseminated infection and mean serum antigen titer are all significantly higher in males than females with cryptococcosis [13]. Currently there is no information on how the immune response to *Cn* differs by gender and why there is a predominance of disease in males.

These observations suggest there is an interaction between the microbe and the male host immune response that results in inefficient control of a *Cn* infection relative to that occurring in female hosts. This hypothesis is supported by the fact that, in our patient set, male AIDS patients have significantly increased risk of death from cryptococcal meningitis despite higher CD4^+^ T lymphocyte counts on admission to the hospital [14], (personal communication). An alternative hypothesis is that microbial factors influence host susceptibility.

In this study these hypotheses were tested by evaluating virulence factor phenotypes of 28 clinical isolates obtained from male and female AIDS patients. Additionally, we evaluated the interaction of *Cn* and human macrophages isolated from healthy male and female volunteers. Virulence factor phenotypes differed between strains isolated from males compared to strains isolated from females. An increase in the release of capsular polysaccharide with the addition of physiological levels of testosterone in both a laboratory and clinical strains were also observed. Finally, male macrophages had poor outcomes after incubation with *Cn* clinical isolates. These results suggest a potential interaction of *Cn* with testosterone that results in increased disease. These results are the first to delineate a possible mechanism for increased incidence of cryptococcal disease in males. Further experiments may advance our understanding of this mechanism and could lead to differential therapies for patients with cryptococcosis.

## Materials and Methods

### Ethics Statement

Venous blood of healthy male and female volunteers was collected in accordance with the guidelines and approval of the Wright Center for Graduate Medical Education Institutional Review Board, Scranton, PA. All blood donors were informed of the goals of the study and agreed by written consent prior to blood donation. All animal use complied with federal regulations and both the University of Utah and Albert Einstein College of Medicine Institutional Animal Care and Use Committee guidelines. The protocol was approved by the Committee on the Ethics of Animal Experiments of the University of Utah (protocol # 97-11011) and Albert Einstein College of Medicine (protocol #20100102).

### Strains

A set of 106 clinical strains isolated from HIV+ patients at the Princess Marina Hospital in Gaborone, Botswana [14] were a kind gift to E.E. McClelland from Drs. Gregory P. Bisson and Rameshwari Thakur. All identifying patient data for these isolates were deleted and unavailable to researchers. To understand why male AIDS patients had increased death, a subset of 28 *Cn* isolates (12 from male patients, 16 from female patients) were used for further characterization. These strains were typed using multi-locus sequence typing [15]. Since eleven of these strains contain one new allele, we are waiting for the MLST curator to assign these strains sequence types. However, comparing the remaining known alleles of these and other strains with the database suggests that all 28 strains belong to either the VNI or VNB groups and are serotype A. While these isolates were originally chosen to be equally matched by patient mortality, the proportion of strains from males and females is very similar to the proportion of male and female patients in the study overall (57% of isolates from females in the subset vs. 60% of isolates from females overall). For all experiments, cultures were started from frozen stocks in order limit effects arising from *in vitro* passaging. The laboratory strain H99 was also used in some experiments.

### Growth Curves/Doubling Time

The growth rate for all clinical isolates was measured as follows. All strains were grown in YPD media at 37°C for 2–3 days. Cells were washed 3× with PBS, counted on a hemacytometer and diluted to 1×10^5^ CFU/ml in YPD media. The baseline absorbance was measured at 600 nm and then the strains were incubated at 37°C. After twelve hours, the absorbance was measured every 3 h until absorbance >1.0 was measured for all strains. The doubling time was calculated using the following formula: Time*(0.693/(Ln(final OD/initial OD))).

### Glucuronoxylomannan Release

To determine if the clinical isolates differed in their ability to release capsular glucuronoxylomanan (GXM) into the medium, capsules were induced in Dulbecco's modified media (DME). Briefly, *Cn* were grown for 2–3 days in YPD broth at 37°C. Cells were washed 3× with PBS, counted with a hemacytometer, adjusted to 1×10^5^ yeast/ml in DME and grown for 18 h at 37°C in 10% CO_2_ [16]. The next day, the DME supernatant was collected and the concentration of GXM in the media was measured by capture ELISA as previously described in [17], except the following mAbs were used in subsequent order: goat anti-mouse IgM unlabeled, followed by mAb 2D10 (IgM), followed by capsule supernatant, followed by mAb 18B7 (IgG1), followed by goat anti-mouse IgG1-AP labeled. The limits of detection for the ELISA using these antibodies are between 0.00005 and 10 μg/ml GXM. The same ELISA was used to determine levels of GXM released in the clinical isolates or the laboratory strain H99 incubated with 400 pg/ml 17-β estradiol (Sigma), 10 ng/ml testosterone (Sigma) and 1/100 dilution 100% ethanol (control). The concentration of GXM was determined relative to known GXM standards on each plate.

### Isolation and culture of human monocytes

Peripheral blood mononuclear cells were isolated by density gradient centrifugation using histopaque-1077 (Sigma). PBMCs were washed with PBS and suspended in RPMI-1640 medium with 10% human serum (50%∶50% male:female, Innovative Research) and 10 ng/ml macrophage colony stimulating factor (M-CSF, PeproTech). Monocytes were allowed to adhere and differentiate into monocyte derived macrophages for 48 hours at 37°C in 5% CO_2_, gently washed and resuspended in RPMI-1640 medium with 10% human sera (50%∶50% male:female) and 10 ng/ml M-CSF for another 48 hours. Macrophages were harvested with Versene (Invitrogen), washed with PBS and resuspended in RPMI-1640 medium containing 10% human serum (50%∶50% male:female) and 100 ng/ml LPS (Fisher). 2×10^4^ macrophages were seeded in 96-well plates and allowed to adhere overnight at 37°C in 5% CO_2_.

### Phagocytosis

The phagocytic efficacy of macrophages isolated from healthy male and female donors was measured as in [18] with the following modifications. Briefly, macrophages were seeded into a 96-well plate (4 wells per *Cn* isolate) in RPMI-1640 media containing 10% human serum (50%∶50% male:female), and 100 ng/ml LPS at a density of 2×10^4^ macrophages and incubated overnight at 37°C with 5% CO_2_. All *Cn* strains were grown for 2–3 d in YPD media at 37°C, washed 3× with 10 ml PBS and stained with 2 µM CMFDA (Molecular Probes) for 30 minutes at 37°C. The strains were washed 2× with 1 ml PBS, counted and opsonized with 10 µg/ml mAb 18B7 and 20% human complement (Innovative Research) for 30 minutes at 37°C before being added to the macrophages in a 1∶1 ratio in RPMI-1640 media containing 10% human serum (50%∶50% male:female), 1 µg/ml LPS (Sigma) and 100 ng/ml human IFN-γ (PeproTech). Macrophages and *Cn* were incubated at 37°C in 5% CO_2_ for 2 hours. After the incubation, the 96-well plate was centrifuged for 5 minutes at 235×*g*, the media was removed and the cells resuspended in 200 µl warm PBS containing 10 μg/ml propidium iodide (Molecular Probes) and 0.01% uvitex 2B (Polysciences, Inc.). Pictures of each well were taken using a Nikon Eclipse Ti inverted fluorescent microscope at 15× magnification with FITC, rhodamine, DAPI and brightfield filters overlaid. *Cn* that were phagocytosed stained green, while *Cn* that were extracellular stained blue. Any dead cells stained red. The number of macrophages containing yeast cells were counted for every well, averaged and reported as percent phagocytosis.

### Macrophage death and *C. neoformans* survival

To determine the susceptibility of male and female macrophages to killing by *Cn* clinical isolates and to measure *Cn* survival after incubation with male and female macrophages, macrophages were seeded into a 96-well plate as in the phagocytosis experiment (above) and incubated overnight at 37°C in 5% CO_2_. All *Cn* strains were grown for 2–3 days in YPD media at 37°C, washed 3× with 10 ml PBS, counted and opsonized with 10 µg/ml mAb 18B7 for 30 minutes at 37°C before being added to the macrophages in a 1∶1 ratio in RPMI-1640 media containing 10% human serum (50%∶50% male:female), 1 µg/ml LPS and 100 ng/ml human IFN-γ. Macrophages and *Cn* were incubated at 37°C in 5% CO_2_ for 1 hour. After the incubation, macrophages were washed 5× with warm PBS to remove extracellular yeast, resuspended in RPMI-1640 containing 10% human serum (50%∶50% male:female) and incubated at 37°C in 5% CO_2_ for 18 hours. After the incubation, the 96-well plate was centrifuged for 5 minutes at 235×*g*, the media was removed (saved in microcentrifuge tubes to measure *Cn* survival) and the cells resuspended in 200 µl warm PBS containing 10 μg/ml propidium iodide. Pictures of each well were taken using a Nikon Eclipse Ti inverted fluorescent microscope at 15× magnification with the rhodamine and brightfield filters overlaid. The number of dead macrophages were counted for each well and averaged to determine the number of dead macrophages.

To measure *Cn* survival, the saved media was combined with washes containing intracellular *Cn*. Briefly, after the pictures were taken to calculate macrophage death, the macrophages were lysed for 5 minutes with 100 µl 0.5% SDS (Sigma). The supernatant was removed and transferred to the same microcentrifuge tube as the removed media. Each well was washed 2× with 100 µl warm PBS and each wash was combined with previous saved supernatants. The total *Cn* supernatant was diluted and plated on YPD plates (2 plates per well). Plates were incubated at 30°C for 48 hours and colonies were counted to determine *Cn* survival.

### Mouse experiments

For the chronic infection experiment, adult male and female BALB/c mice from NCI (>6 weeks old) were infected via intraperitoneal injection with 2.5×10^7^ CFU/ml of strain H99 [19] and sacrificed 39 d post-infection. At the time of organ harvest, there were no obvious signs of clinical disease, though there was some indication that the mice were sick as some had ruffled fur and many had lost 5% of their body weight. The spleens and brains were removed, homogenized, diluted and plated on YPD plates for 2 days at 37°C, after which colonies were counted to determine fungal burden.

For the acute infection experiment, adult male and female BALC/c mice (6 of each per infection, 6–8 weeks old obtained from NCI) were infected via intratracheal injection with 1×10^6^ CFU of one of four clinical strains. Mice were sacrificed at day 7 post-infection and lungs removed and homogenized in 2 ml PBS containing protease inhibitors (Complete Mini; Boehringer Mannheim). Homogenates were diluted and plated on YPD plates for 2 days at 37°C, after which colonies were counted to determine fungal burden. Additionally, lung homogenates were centrifuged at 6000×*g* for 10 minutes to remove cell debris and the supernatants stored at −80°C until tested for cytokine levels.

### Mouse Cytokine ELISAs

BD-OptEA kits (BD Biosciences) were used to assay the lung supernatants for levels of IL-4, IL-10, IL-12, TNF-α and IFN-γ as per the manufacturer's instructions. Lung homogenate was diluted 1∶25 and every mouse lung homogenate was tested in duplicate. The detection limits of the cytokine assays were 7.8 pg/ml for IL-4, 31.3 pg/ml for IL-10, 62.5 pg/ml for IL-12, 15.6 pg/ml for TNF-α, and 31.3 pg/ml for IFN-γ as stated by the manufacturer.

### Statistics

The levels of CD4^+^ T lymphocytes in male and female AIDS patients were analyzed using the non-parametric Wilcoxon Rank Sums test while the increased risk of death in the hospital was analyzed using the chi square test as well as a relative risk with 95% CI and (for adjustment) and odds ratio (which results from logistic regression analyses). Doubling time and GXM release differences were analyzed using the non-parametric Wilcoxon Rank Sums test. Macrophage phagocytosis and death was analyzed using the non-parametric Wilcoxon Rank Sums test while fungal burden within macrophages was analyzed using Analysis of Variance. Mouse fungal burden data was log transformed to achieve normality and analyzed for significance using Analysis of Variance. Mouse cytokine data was analyzed using the non-parametric Wilcoxon Rank Sums test. Power analysis suggested that in order to see a large effect between genders (power level of 0.8, alpha  = 0.05), at least 20 male and 20 female volunteers were needed to donate blood. For all tests, p values <0.05 were considered significant.

## Results

**Figure 1:**
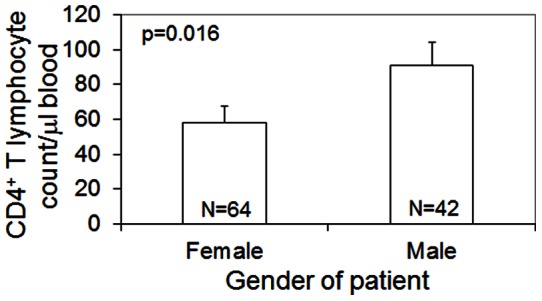
CD4^+^ T lymphocyte counts of Botswanan male and female AIDS patients. Sample sizes are indicated within the bars. Error bars represent standard error of the mean.

To determine if the reported gender differences were perhaps due to disparities in the immune response between genders, we examined immunological parameters from all patients in the study. This revealed that while male AIDS patients with cryptococcal meningitis had significantly higher CD4^+^ T lymphocyte counts upon admission to the hospital (p = 0.016, Figure 1), they had an increased risk of death in the hospital (OR  = 1.8 (0.7−4.9)), even after adjusting for CD4^+^ lymphocyte counts (OR adjusted  = 5.2 (0.9−29), Gregory Bisson, personal communication). This suggests that the male immune response may be less efficient than the female immune response in fighting a *Cn* infection.

**Figure 2:**
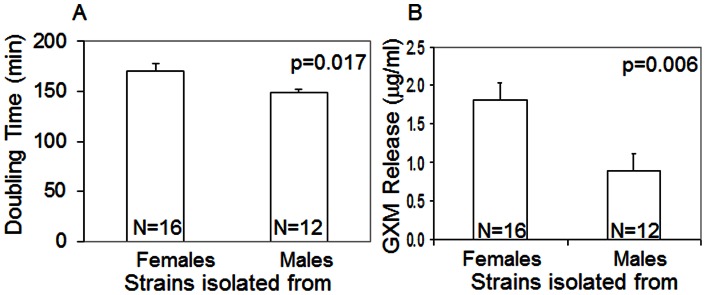
Strains isolated from females have longer doubling times (A) and release more GXM (B). Sample sizes are indicated within the bars. Error bars represent standard error of the mean.

These findings prompted us to characterize the virulence factor phenotypes of 28 clinical isolates. While there was no difference between strains isolated from males and females in melanin production, we found that *Cn* strains isolated from female patients had longer doubling times (170 vs. 148.6 minutes, p = 0.017, Figure 2A), a potential adaptation to an effective host immune response. These strains also released more capsular polysaccharide GXM (p = 0.006, Figure 2B), which may be more efficiently controlled by the female immune response.

**Figure 3:**
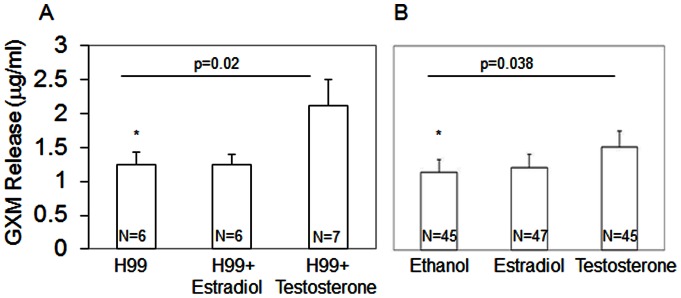
Addition of testosterone increases GXM release in a laboratory strain (A) and in clinical strains isolated from males (B). Sample sizes are indicated within the bars. Asterisks and lines indicate statistical significance. Error bars represent standard error of the mean.

To test whether steroid hormones affected virulence factor phenotypes, a laboratory strain (H99) and the clinical isolates were incubated with physiological levels of 17-β-estradiol (400 pg/ml) or testosterone (10 ng/ml). A significant increase in GXM release was found only after the addition of testosterone for both the lab strain (p = 0.0018, Figure 3A) and the clinical strains isolated from males (p = 0.038, Figure 3B), suggesting a possible microbial interaction with testosterone.

**Figure 4:**
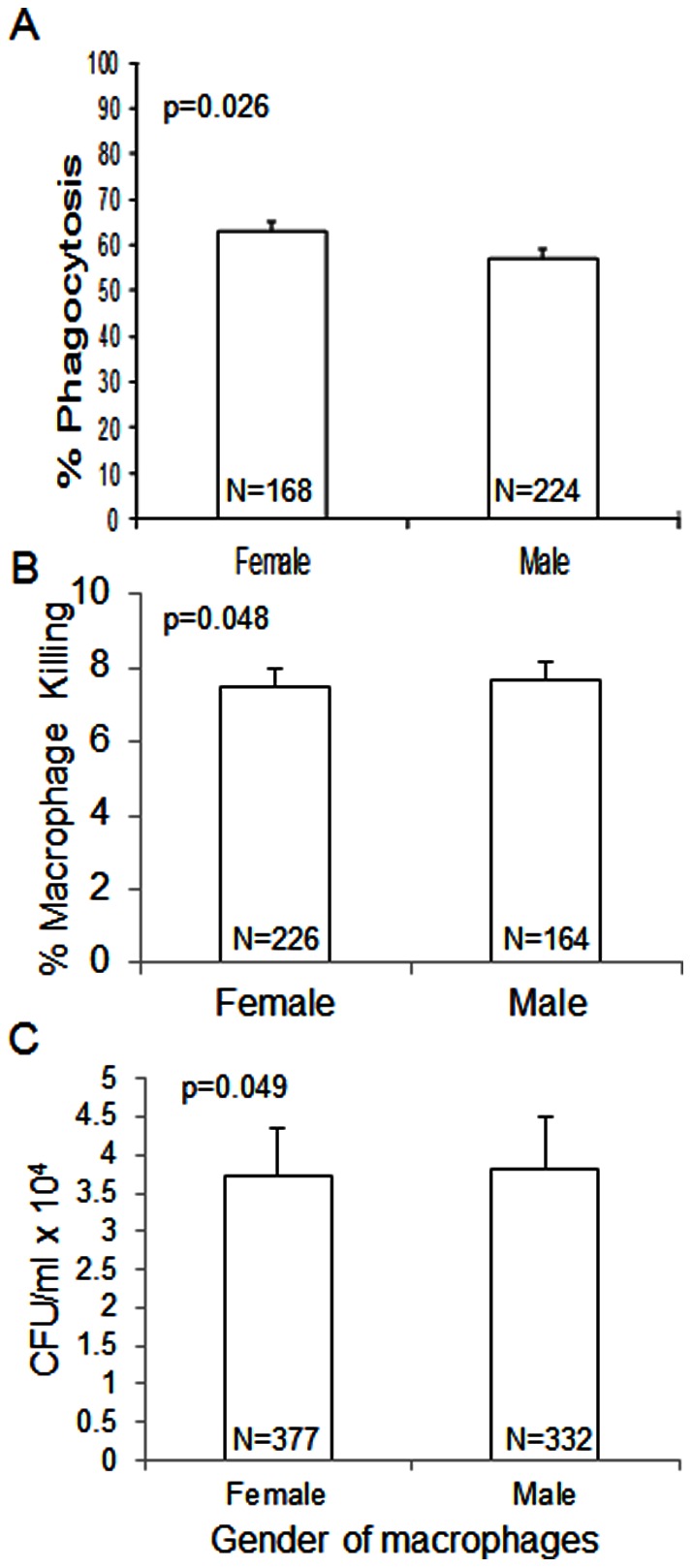
Male macrophages phagocytose less *Cn* (A), have increased death (B) and increased fungal burden (C) compared to female macrophages incubated with *Cn*. Sample sizes are indicated within the bars. Error bars represent standard error of the mean.

To test the interaction of *Cn* with the human immune response, macrophages were derived from peripheral blood monocytes isolated from healthy male and female volunteers. Phagocytic uptake, *Cn*-mediated macrophage killing and macrophage fungal burden were then measured. Female macrophages ingested significantly more *Cn* than male macrophages (p = 0.026, Figure 4A), however, male macrophages were more likely to die (p = 0.048, Figure 4B) and had increased fungal burden (p = 0.049, Figure 4C).

**Figure 5:**
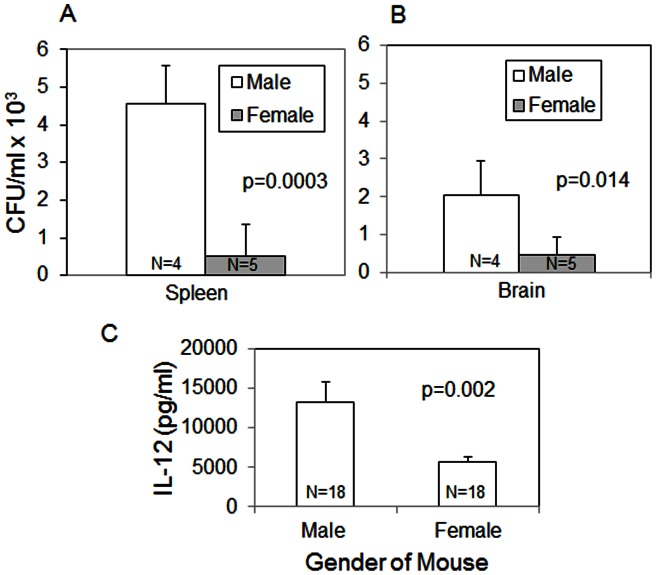
Mouse fungal burden and cytokine levels. Male mice have increased spleen (A) and brain (B) fungal burden during chronic infection and increased levels of IL-12 (C) during acute infection compared to female mice. Sample sizes are indicated within bars. Error bars represent standard error of the mean.

These results correlated with mouse fungal burden experiments, which showed that male Balb/c mice infected intraperitoneally had significantly higher total spleen (p = 0.0003, Figure 5A) and brain (p = 0.014, Figure 5B) fungal burdens than female mice at 39 days post-infection (chronic infection). While differences in fungal burden were apparent at 39 d post infection there was no significant difference in mouse fungal burden in the lungs at day 7 post-infection after an intratracheal injection (data not shown).

To determine if there were immune response differences between male and female mice at day 7 post-infection, concentrations of IL-4, IL-10, IL-12, TNF-α and IFN-γ were measured via ELISA. Levels of IL-4 and IFN-γ were not detected in all mice and there were no differences in concentrations of IL-10 and TNF-α between genders. However, in three out of the four clinical strain infections, male mice had significantly higher levels of IL-12 than female mice (p = 0.002, Figure 5C).

## Discussion

When evaluating an initial subset of clinical isolates for differences in virulence factor phenotypes, we found that there were differences between strains isolated from HIV-infected male and female patients. Further examination of associated patient data found that while male AIDS patients had significantly higher CD4^+^ T lymphocyte counts at the time of admission, they had an increased risk of death during hospitalization. These results suggested that host gender plays a role in *Cn* infection and that the male immune response was less efficient in controlling a *Cn* infection.

In general, men are more susceptible to AIDS and AIDS-related illnesses [20]–[29]. Thus, it is difficult to know whether our data is due to an inherent male gender susceptibility to *Cn* infection or due to a general phenomenon afflicting male AIDS patients. Our finding that the fungal burden is significantly higher in healthy male mice compared to healthy female mice (Figure 5) supports the hypothesis of an inherent male susceptibility to *Cn* infection. Additional data will be required to discriminate between these two hypotheses in humans. It is possible that both processes are influencing the outcome.

To test whether the increased incidence of disease in males [4]–[6] was due to microbial factors influencing host susceptibility or to an ineffective male immune response we evaluated a subset of 28 clinical *Cn* strains for a variety of virulence factor phenotypes as well as how these isolates interacted with macrophages isolated from human male and female donors. Strains isolated from female AIDS patients had significantly slower growth in YPD and significantly higher levels of GXM release than strains isolated from male AIDS patients. These data are supported by the literature, which show that estrogen inhibited growth of *Cn in vitro* [9]. Also, *Cn* strains that grow slowly produce larger capsules [30] and *Cn* cells with larger capsules release more GXM [31]. This data was somewhat counter-intuitive since GXM has been shown to have multiple effects on the host immune response including inhibition of phagocytosis [32], [33], interference with antigen presentation [34], [35] and induction of pro-inflammatory cytokines [36]–[39], among others [40] that would suggest that strains with increased GXM release should be more pathogenic. A possible explanation is the female immune environment selects for *Cn* strains with slower doubling times. Thus, the female immune response would be able to cope with the infection and sequester the GXM released with little damage to the host. It is conceivable that the difference of 22 minutes in doubling time *in vitro* between strains isolated from females and strains isolated from males is biologically significant as *Cn* can fully replicate its DNA or undergo mitosis in 18 minutes [41]. Our data shows the existence of biological differences between *Cn* strains isolated from males and females.

To determine if these phenotypic differences were due to differences in exposure to steroid hormones, we added physiological levels of 17-β-estradiol or testosterone to the clinical isolates and retested for differences in virulence factor phenotypes. The addition of testosterone significantly increased the release of GXM from both a laboratory strain and strains isolated from males. Interestingly, when we included all 28 strains in the analysis, there was only a trend for increased GXM release with the addition of testosterone (p = 0.059, data not shown), suggesting that strains isolated from females release less GXM with the addition of testosterone. Since estrogen does not induce GXM release, only strains that have a higher “native” GXM release will be virulent in females. Testosterone does not induce further GXM release in these strains as they are already near an upper limit of expression. Thus, “weaker” *Cn* strains may be more virulent in males, because testosterone will increase GXM release, increasing virulence. This suggests that *Cn* recovered from humans has been differentially selected by the different gender immune environments and that that there is an interaction of *Cn* with testosterone, but not 17-β-estradiol. These data support recent studies that suggest both the strain and the host contribute to the outcome of *Cn* pathogenesis in humans [1], [2].

We then examined how *Cn* interacted with macrophages from healthy human males and females. In a balanced hormonal environment of 50%∶50% male:female sera, female macrophages phagocytosed significantly more *Cn* while male macrophages had increased death and fungal burden after incubation with *Cn* clinical isolates. We suspect that if we repeated these experiments incubating male macrophages in male sera and female macrophages in female sera, these differences would be even greater. This data suggests that *Cn* replicates more efficiently in male macrophages. This could be due to increased replication or to an inability of male macrophages to kill ingested *Cn*. While further experiments are required to delineate between these two possibilities, this may explain the increased incidence of disease seen in males. It is believed that alveolar macrophages are one of the first lines of defense against a *Cn* infection [42], [43] and that *Cn* replicates inside human macrophages and is then expelled, leaving the macrophage intact [44]. *Cn* is believed to use macrophages as a “Trojan horse” to spread throughout the body and evade immune defenses. If male macrophages show increased fungal burden either due to increased replication or an inability to kill ingested *Cn*, there is a much higher chance *Cn* will disseminate from the lungs to cause fulminant disease.

These data were supported by a chronic *Cn* infection in mice where male mice had significantly increased spleen and brain fungal burden compared to female mice. Interestingly, there was no difference in lung fungal burden between male and female mice during acute infection (day 7 post-infection). The fact that the increased death and fungal burden seen in male macrophages was small, though still significant, may reflect the shortness of the incubation between *Cn* and male macrophages. Our mouse data suggests that a longer incubation time may be required to reflect strong gender-specific differences.

A power analysis was done at the start of these experiments to determine how many male and female volunteers would be needed to see an effect. The power analysis suggested that to observe a large effect (power  = 0.8, alpha  = 0.05), at least 20 male and 20 female blood donors would be required. The fact that these results were observed with only 15 male and 15 female volunteers suggests that the interaction of *Cn* with testosterone is a larger effect than expected, which may help explain the prevalence of disease seen in males.

While these data primarily focus on *Cn*-macrophage interactions, it is conceivable that other aspects of the host immune response may be affected by gender differences and may help explain the increased incidence of cryptococcal disease in males. During a *Cn* intravenous infection in outbred mice using a serotype D strain (52D), females had significantly higher levels of TNF-α and IFN-γ in the spleen and plasma on day 6 post-infection compared to males [45], suggesting that the female immune response was more responsive to a *Cn* infection. Surprisingly, in three of the four acute infections male mice had significantly increased levels of IL-12. These data argue against our hypothesis that the male immune response is less efficient than the female immune response, at least during acute infection, as IL-12 is primarily responsible for stimulation of IFN-γ by both NK cells and CD4^+^ T lymphocytes to activate macrophages to kill ingested microbes [46]. Alternatively, the differences seen in cytokine levels between our acute infections and those seen in Lortholary *et al* [45] could be due to a more robust immune response in female outbred mice. In general, testosterone is thought to decrease the immune response by decreasing antibody production, Fc receptor expression, inducible nitric oxide synthase mRNA expression, and eosinophil degranulation while estrogen increases these immune responses [47]. Besides our data, there have been no studies examining differences in the host immune response between human males and females infected with *Cn*. These types of studies should be given high priority in the future so that researchers can develop a better understanding of how gender-specific differences in the immune response affect a *Cn* infection.

In summary, we have shown that there is an interaction of *Cn* with testosterone that results in increased GXM release and *Cn*-mediated macrophage death. These data suggest a possible mechanism for the increased disease seen in male patients since male macrophages are more likely to be killed by *Cn* than to kill phagocytosed *Cn*. Additionally, these data suggest that the interaction of *Cn* and the gender-specific host immune response is complex and that a prolonged interaction may be required before defects in the male immune response arise. Future experiments to more thoroughly examine this interaction may lead to differential treatment for patients with cryptococcosis.
